# ReNeGate: A Reaction Network Graph-Theoretical Tool for Automated Mechanistic Studies in Computational Homogeneous Catalysis

**DOI:** 10.1021/acs.jctc.2c00404

**Published:** 2022-11-02

**Authors:** Ali Hashemi, Sana Bougueroua, Marie-Pierre Gaigeot, Evgeny A. Pidko

**Affiliations:** † Inorganic Systems Engineering, Department of Chemical Engineering, Faculty of Applied Sciences, Delft University of Technology, Van der Maasweg 9, Delft 2629 HZ, The Netherlands; ‡ Laboratoire Analyse et Modélisation pour la Biologie et l’Environnement (LAMBE) UMR8587, Universite Paris-Saclay, Univ Evry, CNRS, LAMBE UMR8587, Evry-Courcouronnes 91025, France

## Abstract

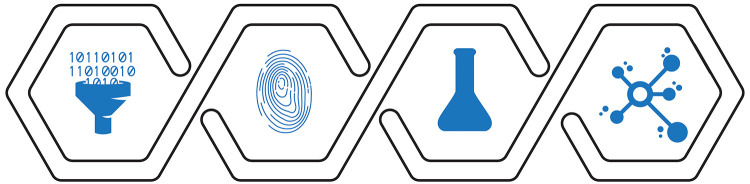

Exploration of the chemical reaction space of chemical transformations in multicomponent mixtures is one of the main challenges in contemporary computational chemistry. To remove expert bias from mechanistic studies and to discover new chemistries, an automated graph-theoretical methodology is proposed, which puts forward a network formalism of homogeneous catalysis reactions and utilizes a network analysis tool for mechanistic studies. The method can be used for analyzing trajectories with single and multiple catalytic species and can provide unique conformers of catalysts including multinuclear catalyst clusters along with other catalytic mixture components. The presented three-step approach has the integrated ability to handle multicomponent catalytic systems of arbitrary complexity (mixtures of reactants, catalyst precursors, ligands, additives, and solvents). It is not limited to predefined chemical rules, does not require prealignment of reaction mixture components consistent with a reaction coordinate, and is not agnostic to the chemical nature of transformations. Conformer exploration, reactive event identification, and reaction network analysis are the main steps taken for identifying the pathways in catalytic systems given the starting precatalytic reaction mixture as the input. Such a methodology allows us to efficiently explore catalytic systems in realistic conditions for either previously observed or completely unknown reactive events in the context of a network representing different intermediates. Our workflow for the catalytic reaction space exploration exclusively focuses on the identification of thermodynamically feasible conversion channels, representative of the (secondary) catalyst deactivation or inhibition paths, which are usually most difficult to anticipate based solely on expert chemical knowledge. Thus, the expert bias is sought to be removed at all steps, and the chemical intuition is limited to the choice of the thermodynamic constraint imposed by the applicable experimental conditions in terms of threshold energy values for allowed transformations. The capabilities of the proposed methodology have been tested by exploring the reactivity of Mn complexes relevant for catalytic hydrogenation chemistry to verify previously postulated activation mechanisms and unravel unexpected reaction channels relevant to rare deactivation events.

## 1 Introduction

Contemporary computational chemistry has reached a stage at which massive exploration into chemical reaction space with an unprecedented resolution with respect to the number of potentially relevant molecular structures is becoming a realistic task. Various algorithmic advances have shown that extensive structural screenings can nowadays be automated and carried out using modern computational chemistry protocols.^1−5^ Automated computational strategies for predicting multistep reaction mechanisms for complex chemical processes, such as pyrolysis, combustion, or catalytic transformations, offer substantial advantages over the conventional strategy largely based on the expert-guided exploration of selected and restricted number of mechanistic alternatives.

Practical catalytic systems are represented by complex mixtures usually containing the catalyst precursor, ligands, solvents, and various additives and promotors next to the substrates and the conversion products. The interactions between these components and their interconversions form large and highly interconnected reaction networks that determine the overall behavior and the performance of the catalytic system. The experimental and computational mechanistic studies aim at identifying the state of the catalytic species and key reaction intermediates, their role in the main catalytic mechanism, and the competing reaction channels toward unselective conversion routes or catalyst deactivation.^6−13^ Such mechanistic insights are critically important for guiding the design and optimization of new and improved catalytic systems in a rational manner.^14−18^

Catalytic reactivity is determined by complex networks of chemical transformations that take place simultaneously or consequently between the different (transient) components of the catalytic mixture. Different stages in a catalytic process, namely, catalyst activation, catalytic cycle propagation, catalyst deactivation, and different nonselective conversion paths, may involve reaction intermediates that are not known *a priori* and will proceed through multiple elementary steps. Even the most advanced experimental operando techniques are not able to capture such a high molecular-level complexity. To establish a comprehensive picture of the catalytic process, computational analysis on such systems requires a thorough exploration of the chemical and configuration space to identify the minima on the potential energy surface (PES) and the pathways connecting them.

The characterization and exploration of the PES is a tedious and challenging task. A conventional workflow in applied computational catalysis studies approaches this task via manual structural explorations, which rely largely on the expert knowledge and a substantial amount of chemical intuition, thus limiting the study to the expected reactivity domains. The last decade has seen a rapid development of various computational approaches to automate the exploration and discovery of complex chemical reaction networks, targeting the reconstruction of a complete atomistic representation of the mechanism of a chemical conversion process.^1−5^ Strategies for the accelerated exploration of reaction networks can vary substantially in the computational costs as well as the comprehensiveness and accuracy of the chemical reaction network that they produce.^19,20^

For example, the global reaction route mapping (GRRM) approach was introduced by Maeda et al.,^21^ in which starting from a given “reactant” configuration, the PES is explored to discover new transition states and intermediates forming the reaction network. Multimolecular reaction paths can be successfully followed using the artificial force-induced reaction (AFIR) method,^21−23^ which directs the transitions from one equilibrium structure to another by applying splitting or merging force to two interacting fragments. This approach was used to automatically construct the catalytic paths for various homogeneous catalytic reactions with transition metal complexes.^24−26^

Despite being highly systematic, such curvature-based exploration strategies may be impractical to studying very large and complex catalytic systems. By introducing principles of algorithmic search, the efficiency of path finding for the conversion of a given substrate state to a defined product state can be substantially improved.^27^ A complementary approach to streamline the exploration of the reaction mechanism is to employ the conceptual knowledge of chemistry. Chemical reactivity can often be well captured by a set of heuristic rules for the transformations that can be applied to graph representations of the molecular system, as successfully demonstrated by Zimmerman and co-workers in their mechanistic studies on organometallic systems.^28−31^ Reiher and co-workers introduced a method based on system-independent heuristic rules,^32^ which was successfully employed to exploit alternative mechanisms of ammonia production with the Schrock dinitrogen–fixation catalyst. Further developments of the method enabled the exploration of transformations involving multiple reactive centers on molecular fragments and/or interactions between different components of the reactive system.^33^

The configuration and reaction space of a molecular system can be directly sampled by solving the nuclear equations of motion in *ab initio* molecular dynamics (AIMD) simulations.^34^ However, considering that even the fastest chemical reactions are rare events, adequate scanning of the reaction space of realistic catalyst systems by the direct atomistic AIMD simulations becomes prohibitively expensive when executed using sufficiently accurate electronic structure methods. The frequency of the reaction events can be greatly accelerated by applying bias potentials that push the system away from the free energy minima along a collective variable (CV), which requires the knowledge of the reaction coordinate and therefore limits the application of this method in exploratory studies.^35−37^ Martínez-Núñez and co-workers introduced an automated procedure called TS search using chemical dynamic simulations (TSSCDS) for the global search of transition states on intermolecular potential energy surfaces based on the PES exploration via the high-energy molecular dynamic simulations.^38−41^ To increase the chances for chemical transformations to occur, the method populates vibrational modes in the system. A similar strategy has also been used to guide the exploration of the configurational space for multinuclear transition metal species in zeolite-based heterogeneous catalysts.^42^ Shannon et al. combined molecular dynamics and statistical rate theory within a ChemDyMe automated mechanism generation method,^43^ in which the search for new reactions is constrained to only the kinetically relevant ones under the specified conditions. Various algorithmic developments in the field have recently been integrated by the group of Reiher in Chemoton 2.0 software that will hopefully make the autonomous mechanistic explorations of complex chemical systems accessible to the wide chemistry community.^44^

Various automated reaction network analysis tools described above enable the automated transition state search and the construction of detailed mechanistic pictures for practical chemical systems. However, the computational demand for such a detailed PES analysis increases exponentially with model system complexity, which reduces the utility of these methods to an exploratory search of secondary transformations (such as nonselective conversion paths, catalyst deactivation, etc.) in extended realistic catalytic systems and/or their integration in high-throughput computational catalyst screening workflows. In this work, we propose a graph-based three-step methodology for exhaustive conformer ensemble exploration and reaction event finding, enabling a comprehensive analysis of complex reaction networks in large molecular ensembles at a reduced computational cost. Here, we employ the CREST^45,46^ method with the systematic root-mean-square deviation (RMSD) biases in terms of pulling factors, which drive the system away from the conformers that have already been explored. The conformer ensembles populated through such parallel metadynamics simulations are then interpreted as molecular graphs and analyzed by the proposed graph-theoretical tools to find unique chemical structures in terms of bonding patterns. The graph theory and computer-based approaches for the analysis of molecular trajectories have proved their value over the last decade in computational chemistry.^47−51^ The main concepts of molecular graph theory, on which the current work is based, are summarized in Section S1 with the common terminology explained in detail in Section S2 of the Supporting Information. A reaction network of such unique chemical species is formed, and the network is further analyzed through inspection of nodes and edges present in the network. The power of the introduced strategy is demonstrated through the analysis of the reaction networks generated for representative model Mn-based homogeneous ketone transfer hydrogenation systems.

The article is structured as follows. In Section 2, we present the description of the new three-step reaction exploration methodology. We present the detailed rationale for the conformer exploration approach introduced to simulate extended molecular systems and a new graph-based tool allowing us to follow the changes in bonding patterns within reactive trajectories to identify reaction events. Section 3 illustrates the capabilities of the developed methodology on three representative case studies of the catalytic and coordination chemistry of Mn(I) compounds. A conclusion section summarizes the presented methodology and obtained results at the end of the manuscript. The additional details of the methodology and the computational results obtained in the validation studies and the full datasets are provided in the Supporting Information. The ReNeGate code is publicly available at: https://github.com/ahashemiche/ReNeGate.

## 2 Automated Reaction Exploration Method

A three-step methodology denoted as ReNeGate is proposed, which is able to automatically handle catalytic systems of arbitrary complexity (multicomponent catalytic mixtures of reactants, catalyst precursors, ligands, additives, and solvents) and is not limited to either predefined chemical rules or predefined reaction coordinates. Reactive space exploration, reactive event identification, and reaction network generation are the main steps taken for understanding the underlying mechanistic pathways in catalytic mixtures. Such a methodology will then be able to comprehensively explore catalytic systems in realistic conditions for either previously observed or completely unknown reactive events. Human bias is sought to be removed in either of the three steps, and chemical intuition is limited to the choice of thermodynamic constraints imposed by applicable experimental conditions in terms of threshold values for allowed transformations.

### 2.1 Reactive Space Exploration

**Figure 1:**
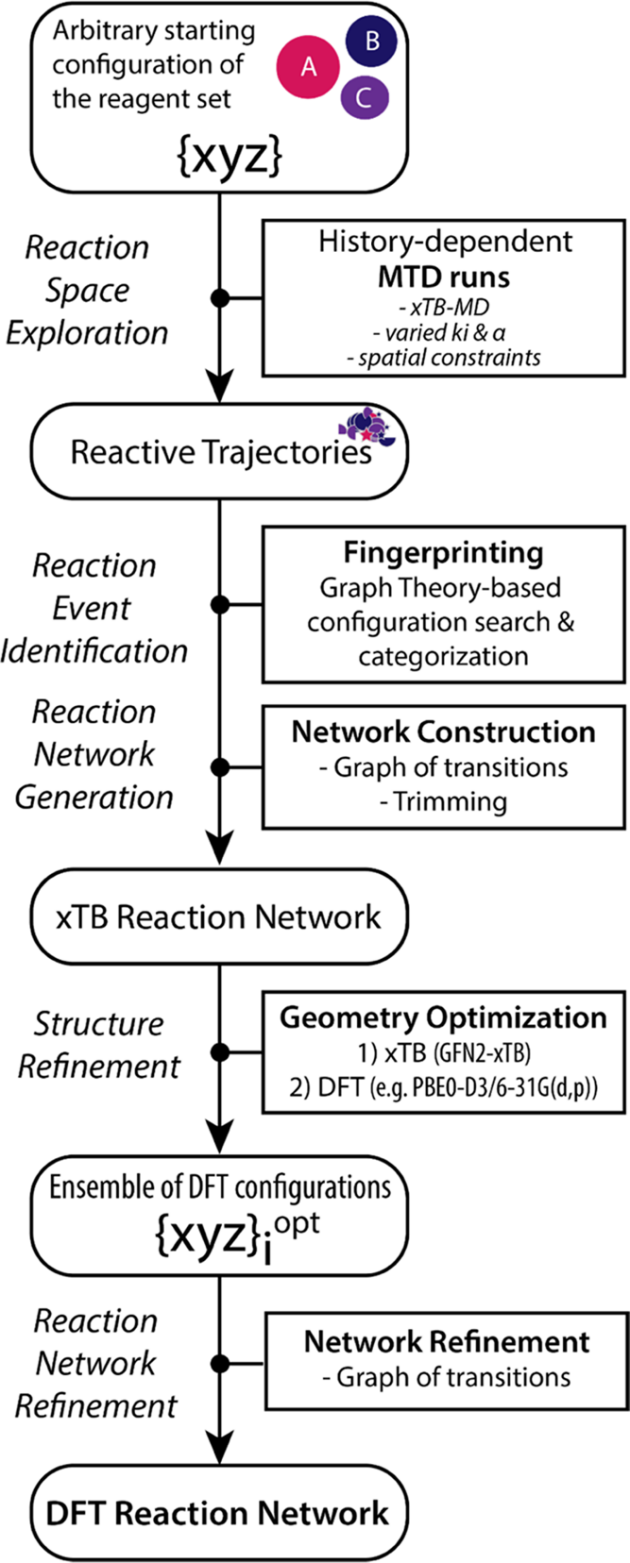
Schematic representation of the ReNeGate workflow involving the sequential reactive space exploration, structural analysis, reaction network generation, and refinement steps.

Figure 1 schematically presents the ReNeGate reaction exploration methodology. The starting point is the exhaustive reaction exploration carried out on a given starting set of reaction components. The identification of unique reactive configurations and reaction states is carried out by analyzing the simulated reactive trajectories in the framework of graph theory. The thus identified reactive states are then refined by geometry optimization at the density-functional theory (DFT) level appropriate for the specific chemical system explored and the final accuracy targeted in the simulations.^10^

Initial reaction space exploration is done using the CREST functionality^46^ in the GFN-xTB^52^ code, where semiempirical xTB-MD calculations with root-mean-square deviation (RMSD)-based metadynamics (MTD) simulations are performed to ensure that the initial reaction space exploration is exhaustive and thorough.^45^ Recent investigations have demonstrated sufficient accuracy of the xTB for high-throughput screening of transition metal complexes,^53^ including Mn(I)-based systems discussed herein as the representative model catalysts.^54^ Imposing RMSD-based metadynamics allows for a thorough exploration of the compound space. The choice of the collective variables (CVs) in MTD simulations is critical, and distinct approaches to this challenging problem in the chemical and biomolecular simulations have been proposed, including diffusion map MD,^55^ targeted MD,^56^ and tabu search^57,58^ methods. Here, we employ the standard root-mean-square deviation (RMSD) in Cartesian space as an unbiased metric as implemented in the CREST functionality in xTB.

Reactive trajectories populated with configurations from the collective MTD simulations from CREST calculations are then analyzed with our dedicated graph-based tool described below for finding unique chemical structures based on bonding patterns. The implementation of such an automated conformer exploration scheme is sought to automate mechanistic studies on the catalytic system of interest and help to reveal unconventional mechanisms and deactivation pathways, which are usually hard to find using conventional expert knowledge-based strategies to mechanistic studies.

### 2.2 Reaction Event Identification and Network Construction

**Figure 2:**
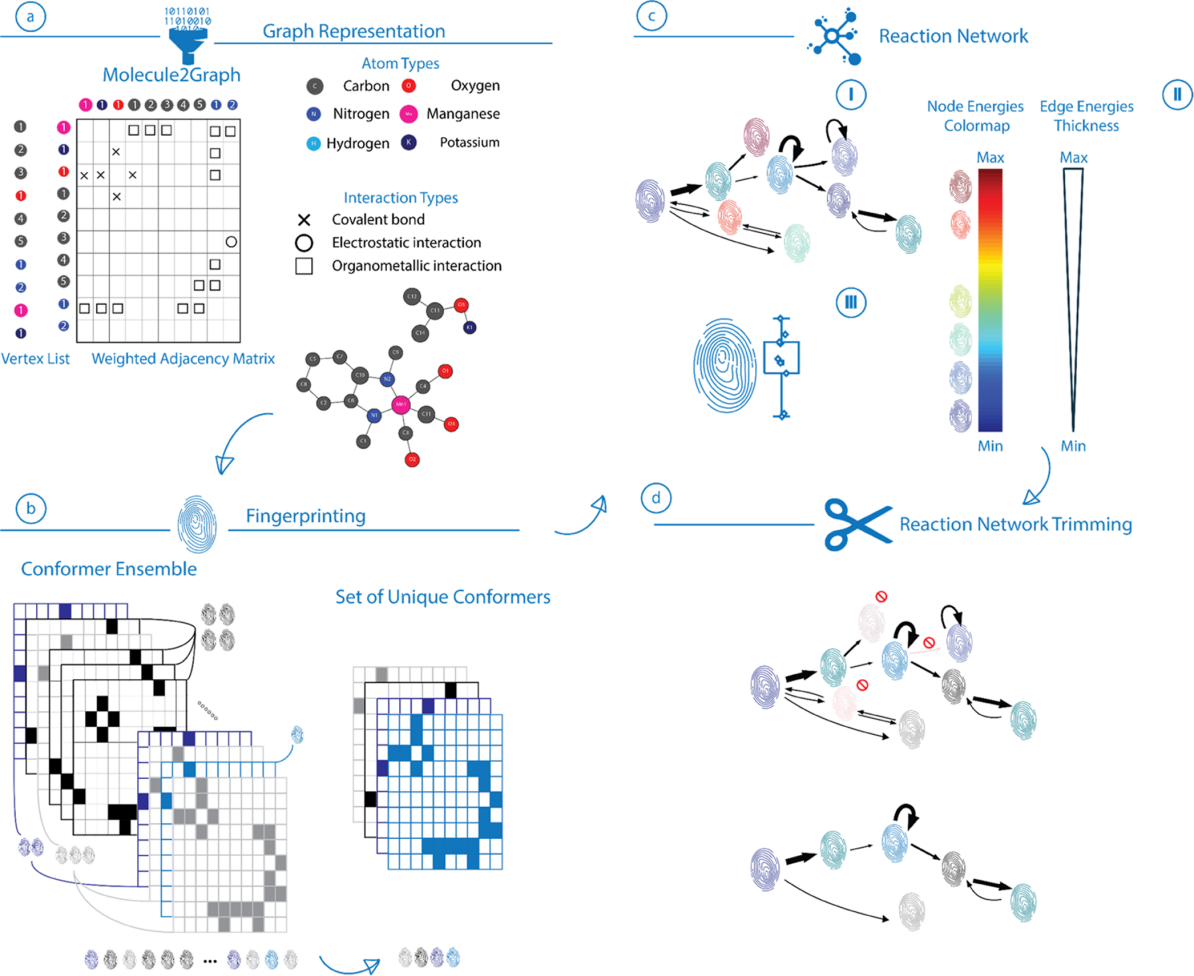
Reaction event exploration scheme following the sequence of (a) the graph representation of the reaction ensemble, (b) fingerprinting of the discovered states, and (c) generation and (d) trimming of the complete reaction network.

Next, we employed graph theory-based algorithms (GTA) to analyze the ensembles of structures produced in the configuration exploration step and to categorize them into “experimentally relevant” ensembles.^59^ The procedure is schematically depicted in Figure 2. The procedure starts with the generation of molecular graph representations for the chemical structures of each given conformer in the graph representation module (Figure 2a).^59^ Next, the conformer ensembles populated in the exploration step are analyzed for fingerprinting and isomorphism check. Further details on the definition of our molecular graph representation and the isomorphism check are given in Section S1.1 of the Supporting Information. A set of unique conformers is identified within the ensemble based on the molecular graphs formed for each conformer (Figure 2b). The combined results of the reaction event exploration and the fingerprinting analysis are then assembled together into the reaction network, in which the specific fingerprints represent nonredundant conformers and the edges represent connections between the conformers in the trajectory (Figure 2c).

Color coding and edge thickness are utilized to visualize energy descriptors for nodes and edges present in the reaction network. The node colors are introduced by the color map (Figure 2c-II) defined based on the lowest (MIN) and highest (MAX) node energies present in the reaction network. The colors for the species are then automatically chosen based on the mapping of the respective node energies. In cases where different isomers are found for a unique conformer, energy for the most stable conformer is used for color coding in the reference graph and variations in energies are visualized as boxplots next to the relevant nodes in energy diagrams. The thickness of the edges present in the reaction network is similarly adjusted by a separate inverse mapping based on the highest and lowest transformation energies, where the transformation energy is defined as the energy difference between the nodes connected with the directed edge. The lowest (most probable) transformation is visualized with the thickest line, while the highest energy transformation has the thinnest edge (Figure 2c-I). Such an analysis allows us to assess the structural flexibility of specific reactive configurations and its relative stability within the reaction network. The final step of the reaction network assembly is the trimming of the network, in which nodes or edge connections with the energies exceeding a predefined energy threshold are removed from the network as schematically shown in Figure 2d. The specific threshold energy value is predefined under the assumption that the states above it have only a minor (if any) contribution to the overall reactivity.

#### 2.2.1 Fingerprinting and Reaction Network Construction

The graph isomorphism tools allow representing each conformer from the screening with a fingerprint molecular graph and comparing it with other species along the simulation trajectory. The fingerprinting of the species within a reactive simulation trajectory proceeds through a sequence of initialization and conformation dynamic analysis steps. During the initialization step, the first snapshot I_1_ of the MD trajectory is read and the first graph G_1_ is defined by identification of the different bonds. Next, the configurational dynamics analysis steps are carried out as follows:

a. Read a new snapshot *I*_i_ and define the associated graph *G*_i_
b. Test if *G*_i_ is isomorphic to *G*_i-1_
c. Else, assign to configurations already identified (update database)
d. Return to step (a) to read the subsequent snapshot.

#### 2.2.2 Reaction Network Trimming

Once a complete reaction network has been formed through the exploration and reactive event identification steps, nodes and edges present in the acquired network (respectively representing chemical species and dynamic connections) are inspected for being accessible within the energy thresholds defined for the system by the user based on the thermodynamic considerations for a given experiment and its representative condition. For all nodes in the graph, the species (nodes) with energies higher than a predefined “node threshold value” together with all respective edges going to and from these nodes are therefore discarded. For all of the edges still present in the network, if the edge weight (representing the relative energy difference between the connected species) is higher than the predefined “edge threshold value”, then edges will be removed from the network. In short, trimming of the obtained reaction networks for the energetically possible pathways is done based on energies of different species and differences in energies for reactive events.

**Figure 3:**
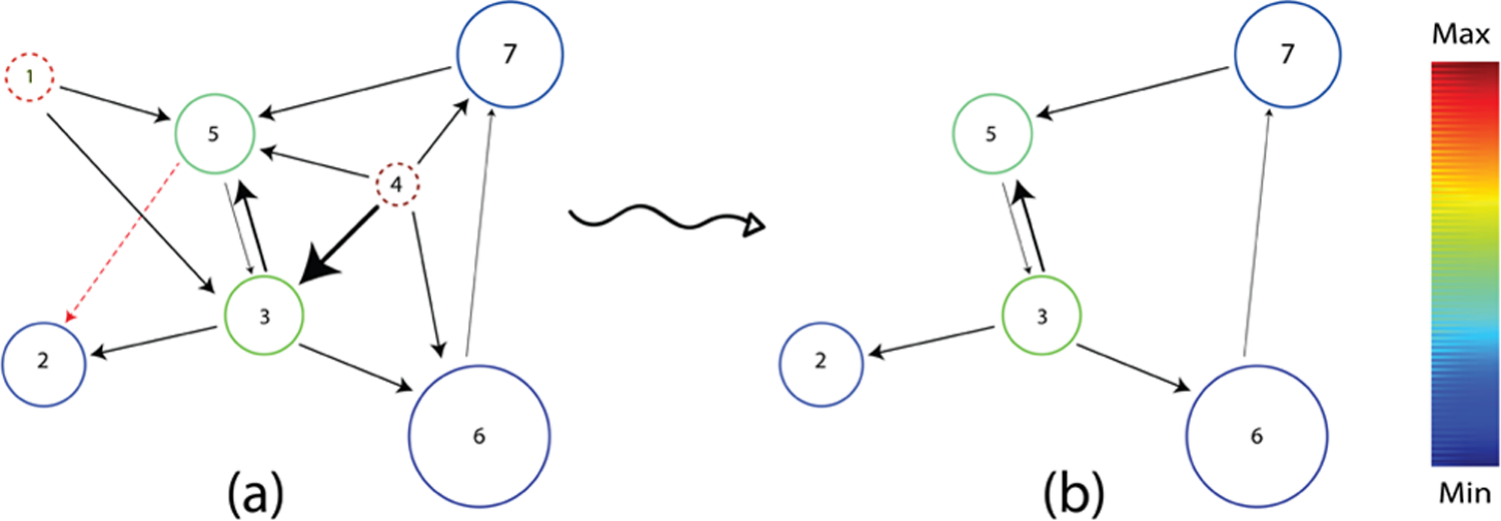
Schematic illustration of an arbitrary reaction network trimming procedure: the original network (a) and the resulting trimmed network (b). Nodes are colored based on the mapping of energies as discussed in Section 2.2. Edge widths are also adjusted based on edge mapping function based on energy differences between the nodes. Arbitrary nodes with energies in both extremes are chosen for clarity. The energies of nodes 1 and 4 (dashed circles) exceed the energy threshold defined for species in this network and are removed by the trimming procedure. The edge connecting nodes 2 and 5 (dashed red line) also exceed the edge threshold value and are hence removed.

Figure 3 schematically illustrates the trimming of an arbitrary chemical reaction network. To facilitate analysis, the size of the nodes in the reaction networks is inversely adjusted by a mapping based on the lowest and highest energies for structures present in the networks. Similarly, the thickness of the edges connecting nodes is adjusted by a separate mapping based on the highest and lowest value for energy differences between the connected nodes. Based on the energies calculated for the nodes present in the arbitrary network, nodes 1 and 4 (shown in dashed circles) have exceeded the predefined node threshold value and the edge connecting nodes 5 and 2 (shown in dashed arrow) has exceeded the edge threshold value. Therefore, these nodes and edges are removed from the original network.

### 2.3 Fragment Analysis

Based on the developments in the reaction network exploration and trimming sections, chemical reaction networks can be built and analyzed for detecting the stable (deactivated) species present on the PES. While such analysis on trajectories including single instances of the catalyst molecule will result in nonredundant unique catalyst fragments, analysis of reaction networks with multinuclear catalytic ensembles is not as trivial. As an extension to the functions described earlier to be able to handle catalytic systems with more than one catalyst molecule, the fragment analysis tool has been developed to be able to provide a list of unique fragments in the cases (1) when changes in the bonding patterns occur in the noncatalyst part of the snapshot or (2) similar catalyst fragments are observed in different unique configurations. To be able to identify unique catalytic events in trajectories populated for systems of arbitrary complexity and to remove expert bias in setting the simulation scenario, the model composition should be considered as close as possible under experimental conditions. Consideration of catalytic systems with more than one metal center introduces new levels of complexity since the algorithms explained in the previous sections should be modified to distinguish different metal centers and enable further comparisons inside a given snapshot (in addition to comparisons within different snapshots).

**Figure 4:**
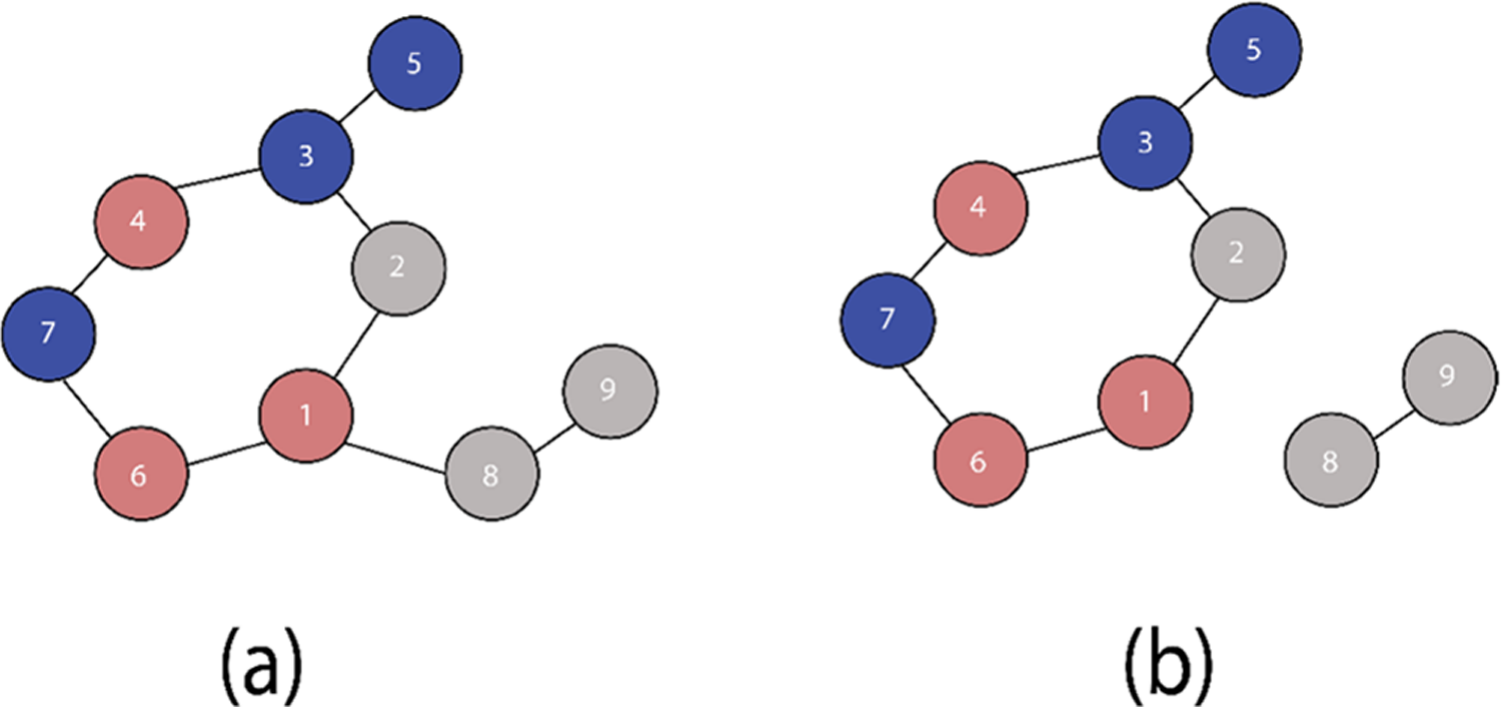
Arbitrary molecular graphs with one (a) and two (b) connected components based on the BFS algorithm.

From a technical point of view, we use Breadth-first search (BFS) algorithm to identify the fragments.^60^ The BFS aims to traverse trees in the graph. It starts at the tree root (on an arbitrary vertex in the graph) and explores all of the neighbor vertices at the present depth prior to moving on to the vertices at the next depth level. Each tree will represent one connected component and each connected component will represent one fragment. Figure 4a,b shows examples of two graphs containing a single and two fragments, respectively.

Once a given trajectory of reactive events is analyzed to identify unique catalyst fragments, a list of connected components is given, which represents the unique set of fragments, including the transition metal (pivot) atom in the analyzed trajectories. Further discussion on the application of fragment analysis tool is given based on a case study presented in Section 3.3, analyzing the possibility of the formation of multinuclear Mn ensembles upon the transformation of two Mn(CO)_5_Br precursors in the presence of an alkoxide base.

## 3 Validation

To assess the performance of the proposed methodology in validating previously observed and identifying unobserved chemical transformations, we applied it to selected representative multicomponent Mn(I)-based (de)hydrogenation catalytic systems. Catalytic (de)hydrogenation reactions promoted by nonprecious 3d transition metal complexes represent more sustainable and environmentally benign alternatives to the established stoichiometric and noble metal-catalyzed processes.^61−65^ Such reactions are of critical importance since they enable efficient transformations of amines, alcohols, and their oxidized counterparts bearing imine, carbonyl, or carboxylate functionalities. Commonly, successful catalytic reactions require the in situ activation of the transition metal complex precatalyst by the reaction with a promotor. Common procedures of catalyst activation in the Mn(I)-based carbonyl reduction systems involve the reaction with an alkoxide base promotor or a hydride donor in the presence of a hydrogen-donating solvent or gaseous H_2_.^66−71^ The selective transformation of the precatalyst complex at this stage is critical for the stability and the overall behavior of the catalytic system.^68^ The formation of undesirable intermediates during the catalyst preactivation may initiate reaction channels giving rise to nonselective conversions and catalyst deactivation. The identification of such minor reaction paths represents a particular challenge both for experimental and computational catalysis studies.

Herein, we specifically aim to utilize the ReNeGate methodology to get an insight into such unexpected reaction paths for representative Mn(I) precatalysts. Two primary case studies are selected, namely, the alkoxide base activation of (I) manganese pentacarbonyl bromide (Mn(CO)_5_Br) catalyst precursor, simulating a widely used protocol for homogeneous catalyst screening with *in situ* catalyst generation^62,72,73^ and (II) cis-Mn(*N*,*N*′-dimethyl-1,2-cyclohexanediamine)(CO)_3_Br (Mn-*N*,*N*) molecularly defined precatalyst.^67,74^ In addition, to demonstrate the potential of the automated fragment analysis, a more complex model capable of capturing interactions between multiple precatalyst species Mn(CO)_5_Br in the presence of the alkoxide activator and BEt_3_ stabilizer toward the formation of multinuclear ensembles is considered with case study III. For these systems, the reaction networks were generated through the conformer exploration, reactive event identification, and trimming steps as implemented in ReNeGate. The optimized structures and the energetics of the intermediates within the produced reaction networks were obtained at the B3LYP-D3/6-31g(d,p) level of theory with empirical GD3BJ-dispersion correction and an implicit SMD model^75^ with the standard parameters for tetrahydrofuran (THF) as a solvent using the Gaussian 16.C01 program.^76^

### 3.1 Case Study I: Mn(CO)_5_Br Precatalyst Activation

**Figure 5:**
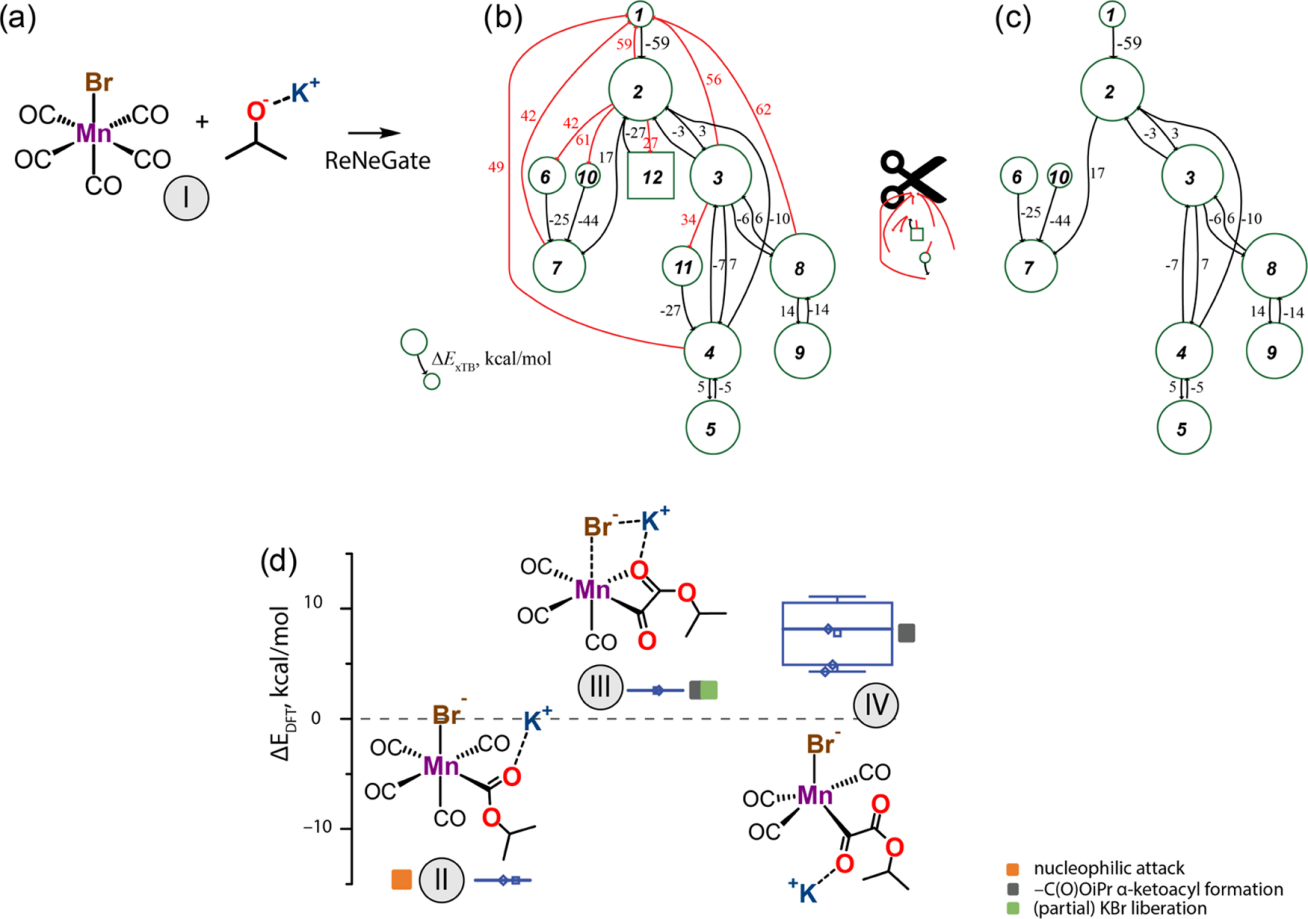
Reaction of (a) Mn(CO)_5_Br and KOiPr resulting in a network of chemical transformations revealed by the ReNeGate method (b) prior and (c) after the trimming procedure. (d) Reaction energy diagram summarizing the distinct product state configurations identified in the network.

For the first case study, we considered the transformations of Mn(CO)_5_Br complex in an alkoxide base solution, simulating a common catalyst activation procedure (Figure 5a). A minimal model containing Mn(CO)_5_Br and KOiPr species was considered here. Parallel metadynamics simulations were carried out using the CREST functionality in the GFN2-xTB method,^77^ where the pushing and pulling strengths (*k* and α) were systematically varied over the parallel simulations. The RMSD difference between structures observed in every trajectory was used to drive simulation away from observing similar structures during the trajectory. Further analysis was done on the basis of ensembles (∼350 structures) populated based on the metadynamics simulations.

Our procedure based on the fingerprinting algorithm yielded a reaction network of unique chemical structures presented in Figure 5b. For the trimming procedure, the edges exceeding the threshold value of +25 kcal/mol (marked with red in Figure 5b) were removed to produce the trimmed reaction network shown in Figure 5c. The procedure also eliminated from the final network the inaccessible nodes after edge trimming (nodes 4 and 12 in Figure 5b) as well as respective connections to prohibited nodes (edges going out from nodes 4 and 12, Figure 5b). Subsequent fingerprinting of the reaction network identified nine distinct species. The structures fingerprinted with similar covalent bonds have been grouped into ensembles of structures and further analyzed for differences in energies and noncovalent interactions. Energy values for the species found for the Mn(CO)_5_Br transformation network are summarized in Table S1 of the Supporting Information.

Stoichiometric reaction with a strong alkoxide base is commonly employed for the activation of a halogen-containing 3d transition metal precatalyst in combination with an acid–base cooperative ligand to a reactive catalytic state accompanied by the liberation of KBr and ligand deprotonation.^62^ Our automated procedure identified highly favorable alternative routes for the reaction of KOiPr base with the Mn(CO)_5_Br precursor, resulting in molecular species more stable by up to 15 kcal/mol compared to the nonactivated state (**I**), representing separate noninteracting alkoxide base and Mn(I) precursor. The main reaction products and their relative stabilities are presented in Figure 5d. Some unique configurations showed substantial structural flexibility, resulting in a range of conformers assigned to a single species and characterized by a range of relative stabilities (e.g., species **IV**). In all routes, the alkoxide nucleophile reacts with the Mn(I)-bound carbonyl ligand. The direct nucleophilic attack results in Mn-acyl complex (**II**). This new reactivity insight has been verified experimentally and inspired the development of new Mn-mediated C–C coupling chemistry recently reported by our group.^78^

In line with earlier experimental studies, the reaction event identification tool has revealed from the reactive trajectories that further migratory insertion of the CO ligand with the −C(O)OiPr α-ketoacyl species is thermodynamically strongly unfavorable.^79^ The resulting C-Mn α-ketoacyl conformers (**III**) and (**IV**) are ca. 5 kcal/mol above the energy of the separate KO^t^Bu and Mn(CO)_5_Br. Due to the minimal size of the model and the lack of explicit solvation, favorable paths toward the KBr liberation were not identified.

These calculations suggested that nucleophiles (e.g., hydrides and alkoxides) could react with a Mn(I)-bound carbonyl ligand, thereby resulting in the formation of Mn formyl or acyl complexes. These findings are in line with prior experimental observations^79−86^ and strongly imply that such reaction paths need to be accounted for when constructing mechanistic hypotheses to rationalize catalytic results based on the in situ catalyst generation protocol, employing the activation of Mn(I) carbonyl precursors in the presence of a strong nucleophile alkoxide base.

### 3.2 Case Study II: Base Activation of a Mn(Br)(CO)_3_-NN Transfer Hydrogenation Precatalyst

**Figure 6:**
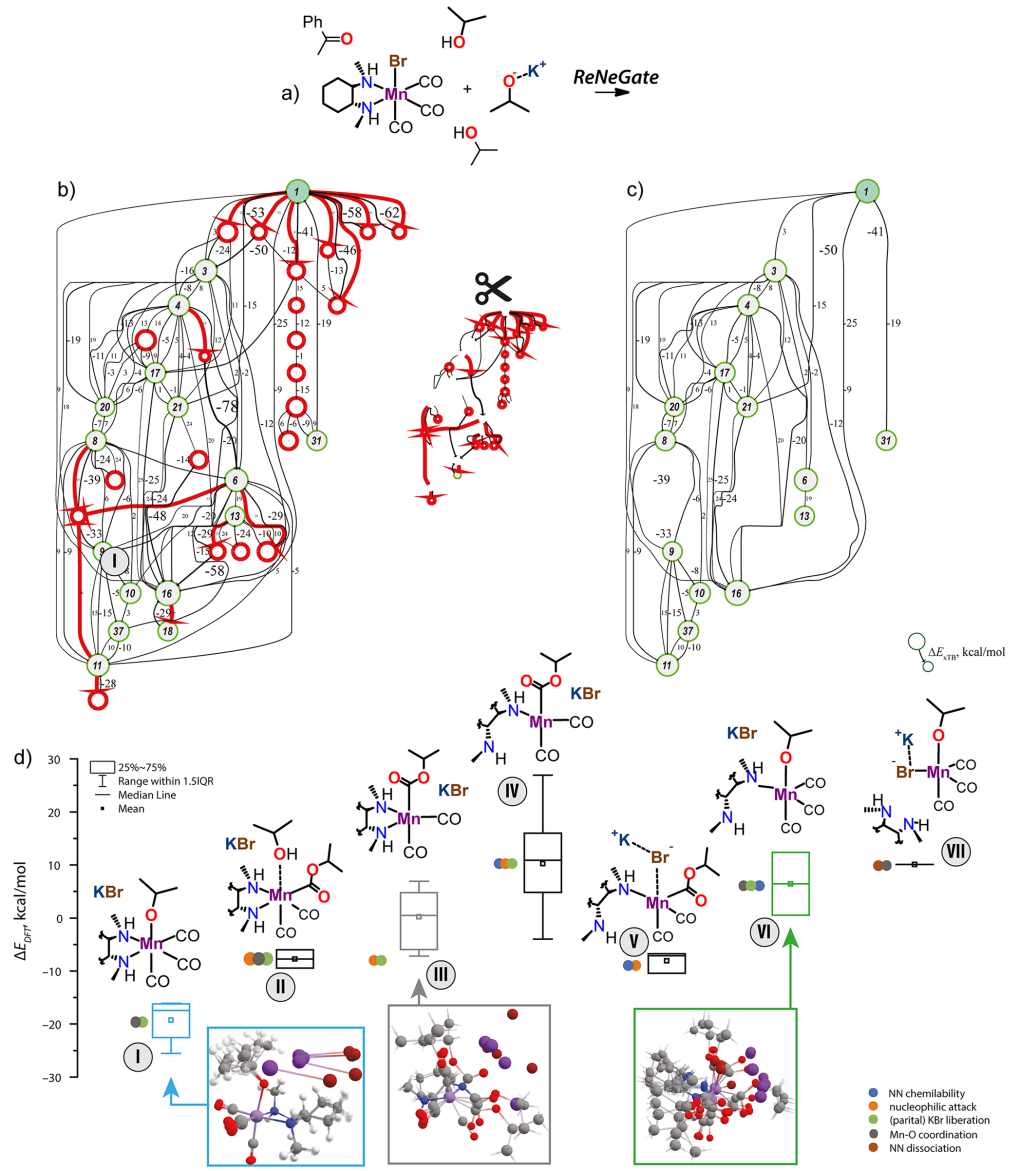
ReNeGate-computed reaction network and the overview of the main potential products of the activation of a model MnBr(CO)_3_NN precatalyst by KOiPr base in isopropanol solvent in the presence of acetophenone substrate. Components of the model system not participating in the reaction are removed for clarity. Panels (b) and (c) show the complete and trimmed reaction networks, while the comparison of the relative stabilities of the identified ensembles of intermediates is summarized in panel (d).

To further evaluate the capabilities of the proposed methodology in exploring the reaction energy landscape for known and unexplored chemistries without additional input from experts, a more complex catalyst system bearing a bidentate ligand has been considered. Specifically, here, we considered the activation of a *N*,*N*′-dimethyl-1,2-cyclohexanediamino manganese tricarbonyl bromide (Mn-*N*,*N*) molecularly defined Mn(I) catalyst precursor with potassium isopropoxide (KO^i^Pr), two isopropanol and one acetophenone molecules (Figure 6a).^67^ Conformer explorations followed by graph-theoretical processing of the metadynamics-based trajectories trimmed down ∼10,000 structures observed in the exploration step at the GFN2-xTB level of theory and further optimization at the DFT level reduced the set of structures to 37 species. These species are the nodes in the reaction network in Figure 6b. They are unique in terms of bonding patterns according to the thresholds defined for covalent and organometallic interactions (see Section S1.1 in the Supporting Information). The structures were further grouped in terms of similar covalent bonds, and the results are summarized in Figure 6d. The distinct chemical states contain a range of isomeric structures with varied stabilities due to the differences in the relative orientations of the components in the model while showing similar covalent bonding patterns and the nature of observed covalent and organometallic chemical changes. The associated energy variations are illustrated as boxplots. The energies of all DFT-optimized species are summarized in Table S2 of the Supporting Information.

The results reveal that the most favorable path for the activation of the Mn-NN precatalyst with an alkoxide base is the ligand exchange reaction, resulting in the rapid elimination of KBr and the formation of Mn-alkoxide complex (**I**) in line with prior mechanistic proposals.^67,74,87^ Such a transformation stabilizes the system by up to 25 kcal/mol compared to the noninteracting components (**I**, Figure 6d). The stability of this state featuring weakly bound KBr and the Mn-alkoxide complex depends on the relative orientation of the molecular fragment. The range of relative energies populated by the different isomers of state **I** (see blue inset in Figure 6d) is shown in Figure 6d with the boxplot. A similar representation is employed for other molecular states discovered computationally.

ReNeGate also identifies a quite unexpected thermodynamically favorable path for the KBr elimination, which is accompanied by a nucleophilic attack of the alkoxide anion by the carbonyl ligand (**II**). Simultaneously, the resulting open coordination site is taken up by an iPrOH solvent molecule explicitly included in the model. This path is less thermodynamically favorable by 10 kcal/mol than the direct ligand exchange reaction. The formation of a 5-coordinated Mn-acyl intermediate (**III**) is slightly less favorable.

The reaction network analysis also identifies reaction channels, resulting in (partial) decoordination of the NN ligand from the metal center (**IV**–**VIII**, Figure 6). While the ligand dissociation resulting in states **VI** and **V** is accompanied by the nucleophilic attack of ^–^OiPr by the Mn–CO moiety, the alternative paths to states **VI** and **VII** result in more conventional undercoordinated Mn-alkoxide and Mn-alcohol adducts. In **VII**, a complete ligand dissociation is observed, whereas in the other three families of intermediates, only one metal–nitrogen coordination is broken. Importantly, only for the case of the Mn-acyl family intermediates (**IV**, **V**), the energy with respect to the free base and Mn precatalyst states is negative. This suggests that the nucleophilic attack by the carbonyl ligand facilitates the ligand decoordination, which may initiate the alkoxide-induced catalyst deactivation observed for Mn(I)-based systems.^66,68,88^

### 3.3 Case Study III: Formation of Multinuclear Ensembles upon the Base Activation of Mn(CO)_5_Br

**Figure 7:**
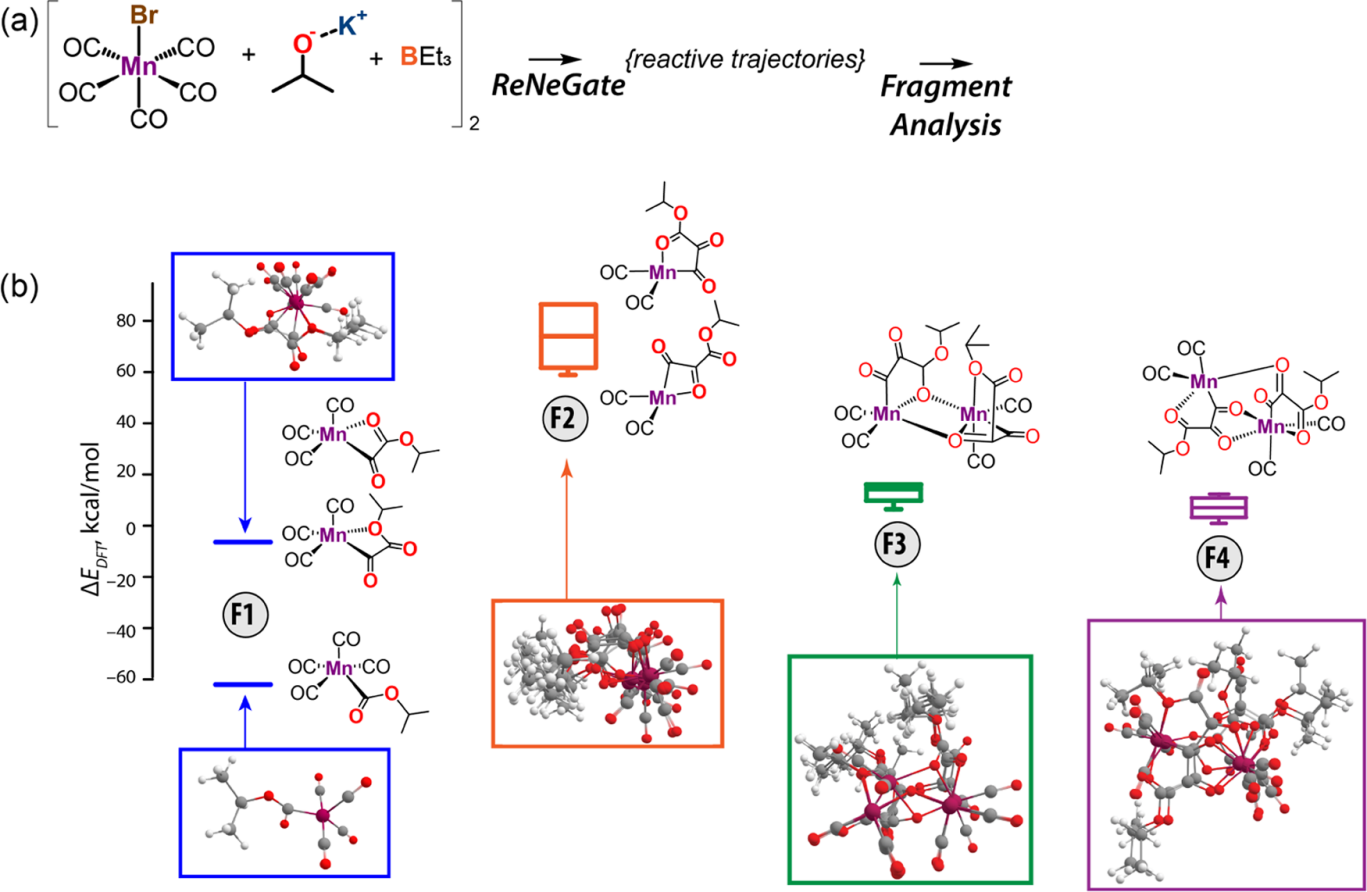
Unique mono- and binuclear Mn complexes identified by the fragment analysis tool in the ReNeGate workflow applied to a system containing two Mn(CO)_5_Br and KO^i^Pr species.

To additionally demonstrate the utility of the fragment analysis tool, we have expanded the case study of the base activation of Mn pentacarbonyl bromide to a hypothetical situation including a more complex reaction mixture including two Mn(CO)_5_Br precursors, KO^i^Pr base, and BEt_3_ stabilizer molecules within the conformer exploration step. The proposed model was built to study the effect of catalyst–catalyst interactions in search for a comprehensive exploration of the PES in realistic reaction conditions and their contribution to stabilization of the reaction intermediates. We have chosen to expand the model system discussed in case study II by introducing an additional catalyst precursor molecule along with a potential BEt_3_ stabilizer, representing a complex experimental reaction environment.^88^ The resulting reactive trajectory was analyzed for finding unique catalytic fragments using the fragment analysis tool within the ReNeGate workflow described herein, with the results summarized in Figure 7. In addition to mononuclear intermediates similar to those observed in case study **I**, we also identified multinuclear complexes, albeit much less stable than the mononuclear Mn-acyl species.

As discussed in Section 2.3, the detection of the fragments and consideration of connections between species is adjusted by the types of interactions (covalent/organometallic/ionic/···) considered for the graph analysis. This means that atoms are considered to be connected if only they have the specified type of interaction. Similar to the previous case studies, the input structure was provided to the ReNeGate workflow, and reaction networks were obtained and trimmed. The species present in the trimmed network were subject to the fragment analysis. Here, covalent and organometallic interactions have been chosen to distinguish different species when following the bonding patterns. Energy values for the species found for fragment analysis are summarized in Table S3 of the Supporting Information. The most stable configurations featured the mononuclear products of the alkoxide attack by Mn–CO to form an acyl complex (F1, lower structure), while further migratory insertion to form C-Mn α-ketoacyl (F1, upper structure and F2 in Figure 7) is thermodynamically unfavorable in line with the chemistry revealed for the trivial model in case study **I**. In addition, di-Mn binuclear fragments have been identified representing the products of the dimerization of the very unstable α-ketoacyl adducts. Such dimers were found to be much less stable than their mononuclear counterparts, suggesting that clustering and aggregation of Mn centers require redox processes not considered within the current models. The binuclear fragments F3 and F4 featured the products of dimerization C-Mn α-ketoacyl adducts (F2). Although the formation of bridging ligands with the various O-atoms of the acyl moiety allowed substantially stabilizing the F2 adducts, the resulting binuclear species were still much less stable than the monodentate Mn-acyl complex. Although in the current case, the increased complexity of the model did not allow identifying new stable configurations, it clearly demonstrates the power of the automated fragment analysis tool. Such straightforward detection of all different catalytic species will become critical when dealing with complex trajectories, where multiple catalytic centers could interact to cooperatively stabilize substrates or such interactions will lead to deactivation of the active center. Changing the type of interactions for analysis (to dynamic hydrogen bonds or ionic interactions) in cases where identification of clusters of hydrogen-bonded structures is of importance will be challenging and can be directly done with the help of the fragment analysis tool.

## 4 Conclusions and Outlook

We described a graph-based reaction network analysis tool for automation of explorative mechanistic studies in homogeneous catalysis. The conformer exploration, reactive event identification, and reaction network analysis are the main steps taken here for understanding the underlying mechanistic pathways in catalytic systems given the reaction mixture as the input. The configurational exploration of the catalytic system is carried out using metadynamic simulations, whose results are interpreted and analyzed in the framework of graph theory to identify reactive events and key intermediates that form a reaction network. Such an initial extensive reaction network is trimmed down to reaction-aware networks through inspection for consistency within energetic thresholds defined for species and transformations. The resulting trimmed networks can be directly used to provide insights into experimental observations or guide the design of further experiments or in-depth computational analysis. Expert bias is sought to be removed in either of the steps and chemical intuition is limited to the choice of thermodynamic constraints imposed by the applicable experimental conditions.

The capabilities of the proposed methodology have been validated for the alkoxide base activation of manganese pentacarbonyl bromide (Mn(CO)_5_Br) and N,N′-dimethyl-1,2-cyclohexanediamino manganese tricarbonyl bromide (MnBr(CO)_3_NN) organometallic complexes commonly employed as precatalysts for (de)hydrogenation conversions. The presented automated reaction network analysis successfully reveals the experimentally observed major reaction channels and also helps identifying the more challenging minor reaction paths, which can be initiated by the catalyst activation procedure and open paths to long-term catalyst deactivation. Specifically, in the case of the MnBr(CO)_3_NN catalyst precursor, the reaction with an alkoxide base, in addition to the desirable ligand exchange producing the catalytic Mn-OR intermediate, gives rise to a number of less favorable reaction channels that can be regarded as the onset of the catalyst decomposition initiated by the nucleophilic attack of the alkoxide anion by the Mn-carbonyl moiety.

There exist several open questions and challenges for further research and expansion of the presented methodology. The entropic effects could be reconstructed based on the conformation energies stored as node attributes for each node present in the reaction network. Threshold values for trimming the reaction networks are decided based on system-specific “reasonable” values by the users. Although this system specificity can be mediated by correlating the trimming values with temperature at which the experiments are usually done for the catalyst not to decompose, it stays as an open question to automatically determine the trimming values based on the chemical nature of the catalytic system. Opportunities exist for using network operations based on the reaction network formalism discussed in the manuscript to describe chemical reactions. This can include but is not limited to: pathway finding operations (starting from/including/leading to specific species present in the network), finding (weighted) shortest paths to identify mechanisms, and finding critical steps (important intermediates) in the network (nodes with large degrees). Such operations and further analysis on networks using machine learning algorithms on the attributes of nodes and edges are also possible for larger reaction networks and are subjects of our on-going studies on automated generation of extended databases of catalytic ensembles.

## Supporting Information Available

Supplementary theoretical details on molecular graph theory for reaction identification; molecular graph theory terminology; supplementary DFT results (PDF)
DFT-optimized structures-Case Study I (XYZ)
DFT-optimized structures-Case Study II (XYZ)
DFT-optimized structures-Case Study III (XYZ)

## Supplementary Material

ct2c00404_si_001.pdf

ct2c00404_si_002.xyz

ct2c00404_si_003.xyz

ct2c00404_si_004.xyz
