# Supplementary material for: ReNeGate: A Reaction Network Graph-Theoretical Tool for Automated Mechanistic Studies in Computational Homogeneous Catalysis
Source: J Chem Theory Comput. 2022 Nov 2;18(12):7470–82. doi: 10.1021/acs.jctc.2c00404 (PMC9753598; doi:10.1021/acs.jctc.2c00404)
Supplement: Supplementary file 1 — ct2c00404_si_001.pdf [file ct2c00404_si_001.pdf]

Supporting Information

ReNeGate: Reaction Network Graph Theoretical tool for automated mechanistic studies in computational homogeneous catalysis

Ali Hashemi[a]*, Sana Bougueroua*[b]*, Marie-Pierre Gaigeot*[b]*, Evgeny A. Pidko*[a]*

[a] Inorganic Systems Engineering, Department of Chemical Engineering, Faculty of Applied Sciences, Delft University of Technology, Van der Maasweg 9, 2629 HZ, Delft, The Netherlands

[b] Laboratoire Analyse et Modélisation pour la Biologie et l'Environnement (LAMBE) UMR8587, Université d'Evry val d'Essonne, Blvd F. Mitterrand, Bat Maupertuis, 91025 EVRY, France

Contents

S1. Molecular graph theory in reaction identification: S3
S2. Relevant Molecular Graph Theory Terminology S7
S3. Total and relative DFT energies of structures identified by ReNeGate analysis S9
S4. Supplementary References.. S12

S1. Molecular graph theory in reaction identification:

Recent implementations of graph theory in chemistry and statistical analysis of MD trajectories have shared a similar degree of granularity in representing molecular systems using simple graphs, where a vertex represents an atom or a molecule and edges represent the interactions/bonds (covalent bond, hydrogen bond…). Developments from Clark *et a l.*, [1-3] Pastor *et al.*, Tenney and Cygan,[4] Choi *et al.*,[5] and Pietrucci *et al.* [6] have been mostly focused on recognizing atomic and molecular cluster geometries during MD simulations (gas phase and liquid phase trajectories), and/or exploring potential energy surfaces of gas phase molecules.[7,8] Presumably the more elaborate graph theory developed in the chemistry and material communities up-to-now was developed by Pietrucci *et al.*,[6] but with the purpose of driving biased MD simulations, which entirely justifies the use of simple graphs. To that end, each vertex of a graph is composed of one atom/one molecule, and the chemical nature of the atoms/molecules is not considered. While these graphs are relatively easy to analyze, they lack specific chemical information (e.g., covalent bonds, hydrogen bonds, exchange of atoms in homogeneous clusters, etc…) that might be relevant for a more detailed characterization of the structures. Furthermore, most of the developed graph theoretical algorithms for chemistry in the literature use adjacency and/or geodesic matrices in order to compare structures sampled over MD trajectories [9], which are not efficient for recognizing identical structures where chemically identical atoms have been swapped, while their ID number in the atoms list keep them non-identical.

S1.1. Definition of a molecular conformation and relationship to graph theory

- **Molecular Graph**

One crucial step of the algorithm is to set up a model that defines a molecular conformation with the right level of granularity. In our case, we have chosen to define the Configuration in terms of covalent bonds and organometallic and ionic interactions formed between the atoms. The definitions are based on *Euclidian distance*s as described below:

- **A bond** is formed between a pair of atoms [a, b] with respective Cartesian coordinates (x_a, y_a, z_a) and (x_b, y_b, z_b), if the Euclidean distance

$$\textit{Equation 1:} \qquad (\sqrt{(xa - xb)^2 + (ya - yb)^2 + (za - zb)^2})$$

  is less than a cut-off distance D_r. For **covalent bonds**, the algorithm defines the D_r distance by the sum of covalent radii of atoms a and b with an additional margin of 2% of this sum. For the **organometallic and ionic interactions**, expert user is given the freedom to set case specific D_r distances, for example, the distance between manganese and oxygen atoms (2.44 Angstrom), etc. This choice was made because the developers assume that the covalent bonds are stronger than the other types of interactions between atoms.

By defining the different bonds, graphs corresponding to different conformations are constructed. By definition, a conformation is translated into a **mixed graph** [10] $\boldsymbol{G = (V, E_C, A_H, E_I, E_O)}$ where [9]:

- V: is the set of all atoms present in the conformation. Each atom represents one vertex in the graph G.
- E_C: the set of **covalent** bonds. Each covalent bond represents **undirected edge** in the graph G.
- A_H: the set of **hydrogen** bonds. Each hydrogen bond represents **directed edge** in the graph G.
- E_I: the set of **intermolecular/ionic** interactions. Each intermolecular/ionic interaction represents **undirected edge** in the graph G.
- E_O: the set of **organometallic** interactions. Each organometallic interaction represents **undirected edge** in the graph G.

  The Cartesian atomic positions taken from the trajectory are used *only* for forming the mixed graphs. Once the graphs are obtained, the changes in the conformations are analysed through the comparison of these graphs using isomorphism check.

- **Molecular Graph isomorphism**

Molecular Configurations are translated into unique graphs based on geometrical threshold values (distance and angles) (Equation 1). Once conceived as graphs, isomorphism check techniques are employed to track possible changes along the trajectory. To reduce the cost of the isomorphism tests, optimisations were used as defining orbits around the atoms [9]. In addition, partitions based on the atom

chemical types of atoms (i.e. oxygen, carbon, manganese, ….) are used to reduce the number of possible permutations in the isomorphism test and thus to reduce the computational costs. For the present work, the algorithm is developed for analysing molecular dynamics trajectories in terms of arbitrary bonding types which represent the conformational change.

Two conformations are different if and only if they are not isomorphic. The isomorphism between two graphs is defined by a *bijection* between them: Two graphs G_a and G_b are **isomorphic** if and only if there exists a **bjection** $\theta_{a,b} : V_a \rightarrow V_b$ such that :

1. $\forall\, v \in V_a, \emptyset(v) = \emptyset(\theta_{a,b}(v)), \theta_{a,b}(v) \in V_b$
2. $[v,u] \in E_{C_a} \Leftrightarrow [\theta_{a,b}(v), \theta_{a,b}(u)] \in E_b$
3. $(v,u) \in A_{H_a} \Leftrightarrow (\theta_{a,b}(v), \theta_{a,b}(u)) \in A_{H_b}$
4. $[v,u] \in E_{I_a} \Leftrightarrow [\theta_{a,b}(v), \theta_{a,b}(u)] \in E_{I\,b}$
5. $[v,u] \in E_{O_a} \Leftrightarrow [\theta_{a,b}(v), \theta_{a,b}(u)] \in E_{O\,b}$

In our case, we apply an isomorphism test considering the atom chemical type as an attribute for different nodes. Consequently, each atom will be given a *colour*. Two atoms can be exchanged if and only if have the same colour. Using this partitioning will not only allow a coherent comparison between graphs but also reduce the number of possible permutations to decide if the graphs are identical or not and thus the algorithm can perform faster. Figure 1 illustrates three example graphs where graphs (a) and (b) are isomorphic, while graph (c) is not isomorphic to a and b.

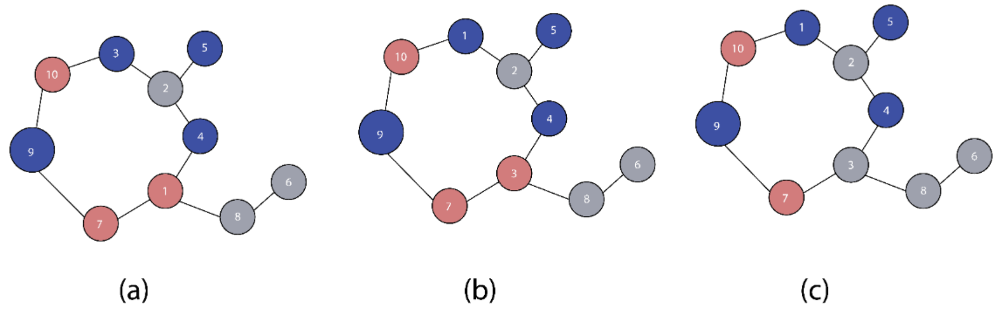

Figure 1 using the isomorphism conditions, graphs a and b are considered isomorphic while graph c is not isomorphic to them

The atomistic granularity level of the simulations is maintained within the graph theoretical analysis proposed here. A molecular system is defined as a graph such that vertices represent atoms of the molecular system and edges represent the bonds formed between these atoms (covalent bond, hydrogen bonds, intermolecular electrostatic interactions, etc., depending on the system). Conformational dynamics of molecular system do occur (evidently depending on internal energies and energy barriers on the potential energy surface), with "fastest motions" being hydrogen/ionic bond dynamics (forming/breaking) along time while larger amplitude motions can induce large structural modifications such as torsional movements. Chemical reactions are defined as the occurrences of formation/breakage covalent bonds, leading to numerous changes in the chemical entities over time. Bond dynamics (forming/breaking) represent, in the graph terminology, a change in the edge sets. The exploration of different Configurations can be seen as an exploration of different graph topologies, tracked using graph theory methods via checking for isomorphic graphs in the Configuration ensemble [11]. An isomorphism between two graphs is a bijection between their vertex sets that preserves adjacency[11], in other words, it is a function between the elements of two sets, where each element of one set is paired with exactly one element of the other set, and each element of the other set is paired with exactly one element of the first set.

Isomorphism checks together with keeping the chemical nature of the atoms are the key components of the *reaction event exploration* step. Isomorphism is used to recognize conformations and construct the graphs of transitions (showing how the conformations are related one to another and the time sequence) for species present in the reference network. The proposed algorithms are applied to trajectories of catalytic mixtures produced in the *Configuration exploration* step. The changes in conformations are followed over time-based scanning trajectories for changes in bonding patterns of choice (among hydrogen bond(s), proton transfer(s), coordination number(s), covalent bond(s) and organometallic interaction(s)).

S2. Relevant Molecular Graph Theory Terminology

- **Graphs**

  A graph **G** is defined as **G = (V, E)**, where **V** represents the set of **vertices** (also called nodes or points) and **E** represents the set of all **edges** (also called links) in the graph. We distinguish many types of graphs:

  - **Directed graph**: a graph **G = (V, A)**, where all the edges are **directed** from one vertex to another. The edges are in general called **arcs**.
  - **Undirected graph**: a graph **G = (V, E)**, where all the edges are **bidirectional.**
  - **Mixed graph**: a graph **G = (V, E, A)** consisting of a set of **undirected** edges **E**, and a set of **directed** edges (arcs) **A**.

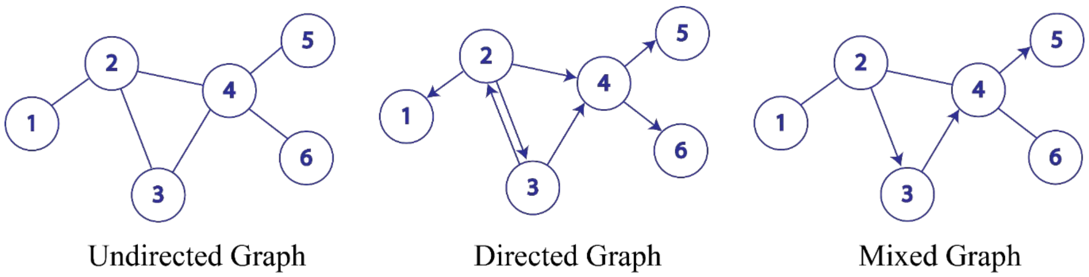

Figure 2 Graph Types based on directionality of edges

  - **Subgraph**: a subgraph **G′ = (V′, E′)** of a graph **G = (V, E)**, is a subset of vertices of **V** and a subset of edges **E**. An **induced subgraph**, is subgraph where the subset of edges contains only the vertices
  - **coloured graph**: it is a graph in which each vertex is assigned a colour. In our case, colours on the vertices represents the chemical types of the atoms.

- **Adjacency Matrices**

  The adjacency matrix is a mathematical way to represent graph. It is an **n×n** matrix where **n** represents the number of vertices of the graph and the values in the matrix represents the edges between these vertices. It is important to note that the matrix representation of an undirected graph is symmetric which is not always the case for a directed or mixed graph.

- **Connected component**

  In graph theory, a connected component of an undirected graph is an induced subgraph in which each pair of vertices is connected to each other via a path.

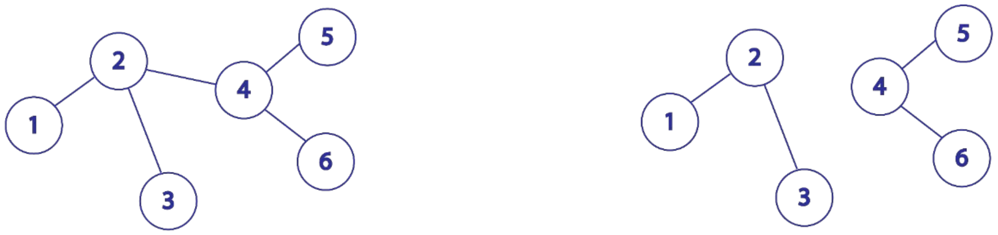

Graph with one connected component

Graph with two connected components

Figure 3 Connected components in graphs

S3. Total and relative DFT energies of structures identified by ReNeGate analysis

Table S1. DFT optimised structures for species present in the ReNeGate -computed reaction network of a model Mn(CO)5Br pre-catalyst by KOiPr base (Figure.4)

Configuration	ID	Energy (Hartrees)	Interaction	Relative Energy (kcal/mol)
Configuration2	II	-5083.214368	Nucleophilic attack	-15.2
Configuration3	II	-5083.214189	Nucleophilic attack	-15.2
Configuration4	III	-5083.180776	C(O)Oipr α ketoacyl +KBr	5.9
Configuration6	II	-5083.214183	Nucleophilic attack	-15.1
Configuration7	II	-5083.213168	Nucleophilic attack	-14.5
Configuration8	IV	-5083.197949	C(O)Oipr α ketoacyl	-4.9
Configuration9	III	-5083.197950	C(O)Oipr α ketoacyl +KBr	-4.9
Configuration10	II	-5083.218841	Nucleophilic attack	-18

Table S2. DFT optimised structures for species present in the ReNeGate -computed reaction network of a model MnBr(CO)3NN pre-catalyst by KOiPr base in isopropanol solvent (Figure.5)

Configuration	Energy(Hartrees)	Interaction	Relative Energy (kcal/mol)
Configuration43	-6055.632111	Mn-OiPr_KBr	0
Configuration4	-6055.630418	Mn-OiPr_KBr	1.1
Configuration31	-6055.624409	Mn-OiPr_KBr	4.8
Configuration39	-6055.620297	Mn-OiPr_KBr	7.4
Configuration32	-6055.618161	Mn-OiPr_KBr	8.8
Configuration40	-6055.617387	Mn-OiPr_KBr	9.2
Configuration62	-6055.617180	Mn-OiPr_KBr	9.4
Configuration33	-6055.617092	Mn-OiPr_KBr	9.4
Configuration48	-6055.606542	PrOH-Mn-OCOR_KBr	16.1
Configuration50	-6055.601041	PrOH-Mn-OCOR_KBr	19.5
Configuration44	-6055.594436	Mn-OPr_N-diss_KBr	23.7
Configuration83	-6055.602944	Mn-OCOR_KBr	18.3
Configuration77	-6055.600940	Mn-OCOR_KBr	19.6
Configuration11	-6055.594466	Mn-OCOR_KBr	23.6
Configuration81	-6055.590699	Mn-OCOR_KBr	26.0
Configuration85	-6055.584681	Mn-OCOR_KBr	29.8
Configuration49	-6055.583848	Mn-OCOR_KBr	30.3
Configuration6	-6055.580477	Mn-OCOR_KBr	32.4
Configuration28	-6055.597800	Mn-Br_N-diss_ROCO	21.5
Configuration88	-6055.596875	Mn-Br_N-diss_ROCO	22.1
Configuration18	-6055.583781	Mn-Br_N-diss_ROCO	30.3
Configuration26	-6055.580434	Mn-Br_N-diss_ROCO	32.4
Configuration87	-6055.579160	Mn-Br_N-diss_ROCO	33.3
Configuration27	-6055.574144	Mn-Br_N-diss_ROCO	36.4
Configuration19	-6055.573722	Mn-Br_N-diss_ROCO	36.7
Configuration23	-6055.571235	Mn-Br_N-diss_ROCO	38.2
Configuration30	-6055.566041	Mn-Br_N-diss_ROCO	41.5
Configuration70	-6055.555067	Mn-Br_N-diss_ROCO	48.4
Configuration17	-6055.548645	Mn-Br_N-diss_ROCO	52.4
Configuration75	-6055.608089	Mn-Br_OCOR_NN-coord	15.1
Configuration54	-6055.602775	Mn-Br_OCOR_NN-coord	18.4
Configuration52	-6055.602211	Mn-Br_OCOR_NN-coord	18.8
Configuration74	-6055.590595	K-Br-Mn-OR_NN-diss	26.1
Configuration45	-6055.571787	K-Br-Mn-OR_NN-diss	37.9
Configuration37	-6055.560253	NN-diss_Mn-Opr_KBr	45.1
Configuration36	-6055.575400	NN-diss_Br-Mn-OiPr	35.6

Table S3: DFT optimised structures for fragments in the ReNeGate -computed reaction network for cluster formation upon base-activation of Mn(CO)5Br pre-catalyst by KOiPr base (Figure.7)

Fragment	Energy(Hartree)	Fragment ID Figure 7	Relative Energy(kcal/mol)
Fragment1	-1911.439826	F1(Lower)	-61.5
Fragment7	-1911.394654	F1(Upper)	-4.6
Fragment6	-1911.394306	F1 (Upper)	-4.1
Fragment5	-1911.394306	F1(Upper)	-4.1
Fragment42	-3822.782028	F4	0
Fragment15	-3822.773267	F3	5.5
Fragment34	-3822.771475	F4	6.6
Fragment35	-3822.771475	F4	6.6
Fragment43	-3822.764056	F4	11.3
Fragment24	-3822.761704	F3	12.8
Fragment29	-3822.76073	F3	13.4
Fragment25	-3822.759259	F3	14.3
Fragment27	-3822.758379	F3	14.9
Fragment17	-1911.345496	F2	57.3
Fragment18	-1911.345226	F2	57.7
Fragment16	-1911.34329	F2	60.1
Fragment20	-1911.343249	F2	60.2
Fragment8	-1911.339866	F2	64.4
Fragment9	-1911.328319	F2	80.0
Fragment40	-1911.324295	F2	84.0
Fragment21	-1911.324019	F2	84.4
Fragment39	-1911.324019	F2	84.4
Fragment38	-1911.324019	F2	84.4

S4. Supplementary References

(1) Mooney, B. L.; Corrales, L. R.; Clark, A. E. MoleculaRnetworks: An integrated graph theoretic and data mining tool to explore solvent organization in molecular simulation. *Journal of computational chemistry* **2012,** *33* (8), 853.

(2) Ozkanlar, A.; Clark, A. E. ChemNetworks: A complex network analysis tool for chemical systems. *Journal of computational chemistry* **2014,** *35* (6), 495.

(3) Mooney, B. L.; Corrales, L. R.; Clark, A. E. Novel analysis of cation solvation using a graph theoretic approach. *The Journal of Physical Chemistry B* **2012,** *116* (14), 4263.

(4) Tenney, C. M.; Cygan, R. T. Analysis of molecular clusters in simulations of lithium-ion battery electrolytes. *The Journal of Physical Chemistry C* **2013,** *117* (47), 24673.

(5) Choi, J.-H.; Lee, H.; Choi, H. R.; Cho, M. Graph theory and ion and molecular aggregation in aqueous solutions. *Annual review of physical chemistry* **2018,** *69*, 125.

(6) Pietrucci, F.; Andreoni, W. Graph theory meets ab initio molecular dynamics: atomic structures and transformations at the nanoscale. *Physical review letters* **2011,** *107* (8), 085504.

(7) Martínez-Núñez, E. An automated transition state search using classical trajectories initialized at multiple minima. *Physical Chemistry Chemical Physics* **2015,** *17* (22), 14912.

(8) Martinez-Nunez, E. An Automated Method to Find Transition States Using Chemical Dynamics Simulations. *J. Comp. Chem.* **2015,** *36* (4), 222.

(9) Bougueroua, S.; Spezia, R.; Pezzotti, S.; Vial, S.; Quessette, F.; Barth, D.; Gaigeot, M.-P. Graph theory for automatic structural recognition in molecular dynamics simulations. *The Journal of chemical physics* **2018,** *149* (18), 184102.

(10) Mohammed, A. M. Mixed graph representation and mixed graph isomorphism. *Gazi University Journal of Science* **2017,** *30* (1), 303.

(11) McKay, B. D. Practical graph isomorphism. **1981**.
